# FAIR and scalable management of small-angle X-ray scattering data

**DOI:** 10.1107/S1600576723001577

**Published:** 2023-03-21

**Authors:** Torsten Giess, Selina Itzigehl, Jan Range, Richard Schömig, Johanna R. Bruckner, Jürgen Pleiss

**Affiliations:** a Institute of Biochemistry and Technical Biochemistry, University of Stuttgart, Allmandring 31, Stuttgart 70569, Germany; b Institute of Physical Chemistry, University of Stuttgart, Pfaffenwaldring 55, Stuttgart 70569, Germany; Argonne National Laboratory, USA

**Keywords:** research data management, FAIR data principles, small-angle X-ray scattering, SAXS, lyotropic liquid crystals, alkyltrimethylammonium surfactants, phase diagrams

## Abstract

A modular research data management toolbox based on the programming language Python, the widely used computing platform *Jupyter Notebook*, the standardized data exchange format for analytical data (AnIML) and the generic repository Dataverse has been established and applied to analyze small-angle X-ray scattering (SAXS) data according to the FAIR data principles (findable, accessible, interoperable and reusable). The *SAS-tools* library is a community-driven effort to develop tools for data acquisition, analysis, visualization and publishing of SAXS data. Metadata from the experiment and the results of data analysis are stored as an AnIML document using the novel Python-native *pyAnIML* API. The AnIML document, measured raw data and plots resulting from the analysis are combined into an archive in OMEX format and uploaded to Dataverse using the novel *easyDataverse* API, which makes each data set accessible via a unique DOI and searchable via a structured metadata block. *SAS-tools* is applied to study the effects of alkyl chain length and counterions on the phase diagrams of alkyltrimethyl­ammonium surfactants in order to demonstrate the feasibility and usefulness of a scalable data management workflow for experiments in physical chemistry.

## Abstract 2

A modular and extensible research data management toolbox based on the programming language Python and the widely used computing platform *Jupyter Notebook* has been established for the acquisition, visualization, analysis and storage of small-angle X-ray scattering data.

## 1. Introduction

Efficient data management is considered to be a key component for the digitization of chemical research, because limited data accessibility, poor data quality and data gaps hamper the transition to sustainable chemistry (Fantke *et al.*, 2021 ▸) and prevent the wide application of data science methods in chemistry (Artrith *et al.*, 2021 ▸). However, research data management in chemistry is still in its infancy. Initiated by the claims of a 2005 essay (Ioannidis, 2005 ▸), the continuing debate on the existence of a reproducibility and replicability crisis in scientific research in general (Sayre & Riegelman, 2018 ▸), and in chemistry specifically (Han *et al.*, 2019 ▸; Gibb, 2014 ▸; Coudert, 2017 ▸), demonstrates the critical role of reproducible and accessible scientific data for the credibility of research results.

The FAIR guiding principles for scientific data management and stewardship (findable, accessible, interoperable and reusable; Wilkinson *et al.*, 2016 ▸) provided the groundwork for sustainable research data management (RDM), which has since become promoted, encouraged and sponsored by various initiatives, consortia and organizations, such as the German National Research Data Infrastructure with consortia focused on chemistry (NFDI4Chem; Herres-Pawlis *et al.*, 2020 ▸; Steinbeck *et al.*, 2020 ▸) and catalysis-related sciences (NFDI4Cat; Wulf *et al.*, 2021 ▸), the European Commission with its Horizon 2020 (European Research Council, 2017 ▸) and Horizon Europe (Directorate-General for Research and Innovation, 2021 ▸) funding programs, and the IUPAC (McNaught & Wilkinson, 2019 ▸). Through the combined efforts of individuals, institutions and programs, a multitude of standards, platforms, formats and tools have become available: repositories (Crosas, 2011 ▸), databases (Kearnes *et al.*, 2021 ▸), electronic laboratory notebooks (Tremouilhac *et al.*, 2020 ▸), standardized data exchange formats (Range *et al.*, 2021 ▸) and ontologies (Hastings *et al.*, 2016 ▸).

The efficient management of research data is widely seen as key to the digitization of chemical research (Wulf *et al.*, 2021 ▸), and the guiding principles of FAIR data are generally accepted as a necessary condition for the reproducibility and efficiency of research (Wilkinson *et al.*, 2016 ▸). However, the implementation of good research data management is still a challenge (Shearer, 2015 ▸). Interestingly, the majority of studies focus on ‘giving access to data’ and ‘preserving data’, whereas the aspects of ‘data analysis’ and ‘processing data’, which are most relevant in everyday laboratory practice, are still under-represented (Perrier *et al.*, 2017 ▸). In order to integrate these resources into the complex workflows used in chemical research, they have to be extended and adapted to everyday laboratory practice. Chemists routinely use a multitude of tools for data collection, analysis and visualization, which comprise proprietary software by instrument manufacturers for data collection, as well as software such as *Excel* (Microsoft), *Origin* (OriginLab) or MATLAB (Mathworks) for analysis and visualization.

The second challenge is the selection of a suitable data format for the reporting of experiments and the exchange of data. The FAIR principles implicitly require standardized data exchange formats, which are the basis of interoperability and reusability. A multitude of different data exchange standards are available for reporting various types of data, but their feasibility and usefulness are largely dependent on the purpose for which these standards were designed. It is also important to note that standardization and openness themselves are far more important than the choice of a specific standard, because interoperable data can be readily transferred from one format to another. Some noteworthy standards are HDF5 (The HDF Group; https://www.hdfgroup.org/solutions/hdf5/), which excels at handling extraordinarily large data sets, JCAMP-DX (McDonald & Wilks, 1988 ▸), which is the IUPAC standard format family for spectral data exchange, and MatML (https://www.matml.org/), which is an XML-based format for exchange of material specifications.

While these formats offer unique advantages and strengths, none is particularly well suited for small-angle scattering (SAS) data. Two standards stand out for application in SAS experiments. NeXus (Könnecke *et al.*, 2015 ▸), which is built on HDF5, was specifically developed for capturing raw and processed data from X-ray, neutron and muon experiments. The Analytical Information Markup Language (AnIML; https://www.animl.org/) was developed as a generic chemical standard for FAIR reporting of any kind of analytical data (Schäfer *et al.*, 2004 ▸). So far, AnIML has been shown to be a useful tool for standardized storage of data from UV/Vis spectroscopy and chromatography (Fiege, 2007 ▸).

In this paper, we describe the implementation of AnIML to store one-dimensional small-angle X-ray scattering (SAXS) data, a novel and community-driven toolbox, *SAS-tools*, for data acquisition, analysis and visualization of SAS data, and three newly developed APIs, *pyAnIML*, *easyDataverse* and *pyDaRUS*, which mediate the conversion of raw data in PDH format (generated by the instrument, SAXSess mc^2^, Anton Paar) to AnIML, the integration of tools for data analysis, and the upload of AnIML data sets and their metadata to the Stuttgart Dataverse installation DaRUS. The workflow was implemented as *Jupyter Notebooks* (https://jupyter.org), because they provide the modularity, high flexibility and customizability of a widespread platform, while at the same time being open, lightweight and transferable.

To demonstrate the usefulness of this workflow, we recorded the phase diagrams of two water/surfactant systems, monitoring the structural changes by SAXS measurements with varying concentration and temperature. The surfactants are members of a well known family of cationic surfactants, whose best-known representative, cetyltrimethylammonium bromide (C_16_TAB), features a hexadecyl chain. The first phase diagram of C_16_TAB with water dates back to 1960 (Husson *et al.*, 1960 ▸), and it was later established in more detail (Wolff & von Bünau, 1984 ▸; Wärnheim & Jönsson, 1988 ▸). Phase diagrams of the shorter homologs (C_14_TAB, C_12_TAB and C_10_TAB) were identified, with the tendency of phase boundaries to shift at higher surfactant concentrations (Wärnheim & Jönsson, 1988 ▸; McGrath, 1995 ▸; Varade *et al.*, 2008 ▸). The lyotropic liquid crystalline (LLC) phases of C_8_TAB (octyltrimethylammonium bromide) have not been investigated in detail so far, so only simplified phase diagrams of the system with water exist, which exhibit a single liquid crystal phase without structural identification (Chen & Hall, 1973 ▸; Barker *et al.*, 1974 ▸; Fukada *et al.*, 1998 ▸). The surfactant series has also often been used to study the effect of the counterion (Lima *et al.*, 2013 ▸; Liu & Warr, 2014 ▸). Several publications have stated rather ambiguous or even contradictory results (Broome *et al.*, 1951 ▸; Balmbra *et al.*, 1969 ▸; Soederman *et al.*, 1985 ▸; Edlund *et al.*, 1997 ▸). For cetyltrimethylammonium chloride (C_16_TAC) two intermediary phases were found (Blackmore & Tiddy, 1988*a* ▸, 1990 ▸) and assigned (Henriksson *et al.*, 1992 ▸). However, a later identified phase diagram of the same surfactant did not feature these phases, even though the authors applied polarized optical microscopy and SAXS measurements in an effort to establish them (Chen *et al.*, 2012 ▸). We are not aware of any detailed phase diagrams published for any of the other homologs of the C*_x_* TAC series.

Due to the complexity of the phase diagrams discussed above, it is clear that a phase assignment by polarizing optical microscopy does not suffice for such systems, but instead structure-determining methods such as SAXS or small-angle neutron scattering (SANS) are required. Some contradictory results reported in the literature could be resolved by a consistent re-analysis of published data. However, raw data including the exact measurement points (concentration and temperature for every data point) are rarely given in scientific reports, which considerably hampers progress in this research field. The same applies for the interpretation of raw data, which may be ambiguous in some cases. Access to raw data would not only clarify apparent contradictions in different publications but also facilitate the comparison of different data sets when investigating the influence of the alkyl chain length or counterion on the observed phase behavior. The prerequisite, however, is that the data are not only accessible but also readable and manageable for a broad scientific community over the long term.

## 2. General organization of the RDM platform for SAXS data

To demonstrate the feasibility of a bottom-up approach to RDM which is immediately applicable and scalable, we developed an extensible RDM platform for working with SAXS data. It uses AnIML (Schäfer *et al.*, 2004 ▸) as a standardized data exchange format, *Jupyter Notebook* for the implementation of workflows, and Dataverse (https://dataverse.org/) as a generic platform for the exchange and findable publication of data and metadata. The RDM platform comprises tools for conversion, visualization and analysis of SAXS data, application programming interfaces for AnIML and Dataverse, and an example workflow for investigating phase diagrams of lyotropic liquid crystals.

### 2.1. Dependencies

Python 3.10.6 was used as the scripting language throughout this work. Miniconda3 (https://docs.conda.io/en/latest/miniconda.html) was used to manage virtual environments. The following third-party packages and libraries (including their respective dependencies) were used: *black* 22.12.0, *coverage* 7.0.0, *ipykernel* 6.9.1, *lmfit* 1.1.0, *lxml* 4.9.2, *matplotlib* 3.6.2, *numpy* 1.23.5, *pandas* 1.5.2, *pytest* 7.2.0, *python-libcombine* 0.2.19, *scikit-learn* 1.2.0, *scipy* 1.9.3, *seaborn* 0.12.1, *twine* 4.0.2 and *versioneer* 0.28.

As is customary, *Jupyter Lab* and the *nb_conda_kernels* 2.3.1 package were installed into the *base* environment of the system to make all virtual environments containing an *ipykernel* 6.9.1 package available as kernels from this base *Jupyter* instance. The actual production environment *fairsaxs* served as kernel for all *Jupyter Notebooks* presented in this work and contained all the packages listed above. The complete requirements for both the *base* and *fairsaxs* environment can be found in their respective conda environment YAML files (Table S1 in the supporting information).

### 2.2. SAS-tools

#### 2.2.1. Readers

The *readers* package within the *SAS-tools* library contains tools for data acquisition and reformatting, as well as utility functions like type inference. The *PDHReader* class within the *pdhreader.py* module was developed specifically for parsing data and metadata from the ASCII-encoded raw data sets in PDH format, which is one of the formats implemented by Anton Parr for their SAXess products. The class has methods for enumerating available PDH files within a given directory and separately extracting data as *pandas.DataFrame* and metadata in XML format as *lxml.etree.ElementTree* from these files. The *OriginReader* class of the *originreader.py* module is an *ad hoc* tool for the acquisition of curve fitting results from *Origin* output files in TXT format and conversion of these data to *pandas.DataFrame* for downstream use. To work more conveniently with data contained in AnIML documents, a special *SeriesReader* (in *seriesreader.py*) was developed to parse exclusively for *Series* elements within an AnIML document. Using the *pyAnIML* API described in detail below, an AnIML document object is searched for its *Series* elements contained in any *SeriesSet* or *Category* and their unique *seriesID* attributes are returned. Any of these *Series* can then be converted to a *pandas.DataFrame* after being selected by its *seriesID*. The utility function *infer_type* which is found in the module *infer_type.py* can be applied when writing data to AnIML, making it easy to select the correct corresponding AnIML data type from its Python data type.

#### 2.2.2. analyzer

The *analyzer* package of *SAS-tools* includes modules for analysis and visualization of SAS data. It currently contains tools for general curve fitting using Lorentzian, Gaussian or Voigt functions, as well as for working with one-dimensional SAXS of lyotropic liquid crystals. Peak fitting is performed by the *CurveFitting* class contained in the *curvefitting.py* module. With the help of the *find_peaks_cwt* method based on the Python library *signal*, the raw data provided as a *pandas.DataFrame* can be searched for peaks of different widths. Starting from the estimated peak positions, the initial parameters for the fit can be set manually or automatically (*set_specifications_manually* or *set_specifications_automatically*, respectively). In automatic mode, the number of models used for the fit and their initial parameters (initial guess) are determined by the number and positions of the peaks found, respectively, so that each peak found is fitted. In manual mode, initial parameters and model types must be specified manually for each peak to be fitted. To ensure reproducibility of the curve fitting, all parameters used for the fitting process are stored in a .json file named models_dict_filename. It contains a list whose elements represent the single models. Each of these individual models is defined by a model type, the position of the center of the model, its amplitude, its width (sigma) and optional help parameters, which represent a lower and upper bound to which the model’s center position is restricted during the fitting process. Thus, given these parameters, rerunning the fitting process will always yield the same fitting results.

**Figure 1:**
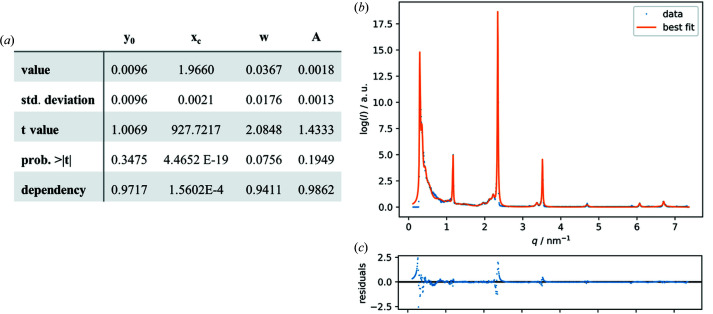
Fitting of measured scattering data of cholesteryl palmitate. (*a*) An excerpt from a TXT file with fitting data obtained from a Lorentzian fit in *Origin*. (*b*) Fitting by the *curvefitting* module (27 models of the Lorentzian type). (c) Residuals of the fitted curve.

The actual fit is performed by the *fit* method, which is based on the Python library *lmfit*. It yields an *lmfit model_result* object that contains all the information about the fitted models and is stored in a file named model_result_f ilename.sav. It can be used to plot the result, as shown on the right-hand side of Fig. 1 ▸, or for further analysis. The *save_list_of_peak_centers* method stores the positions of the peak centers of the fitted models in a TXT file. Additional methods allow for plotting the raw data, as well as individual output data like the peaks found or the fitting results. The main classes *PrepareStandard* and *LLCAnalyzer* are contained in *saxsanalyzer.py*. To be able to calibrate measured SAXS data against a standard, *PrepareStandard* can be used to calculate the linear regression of the measured scattering vectors against the literature-known scattering vectors of the SAXS standard used. The user has the choice either of selecting a pre-defined standard from the *SAXSStandards* enum found in the *enums.py* module, which automatically sets the corresponding literature-known scattering vectors, or of directly providing these literature-known scattering vectors or calculating them from reported lattice-plane distances. The tuple of slope and *y*-axis intercept can then be used in the *LLCAnalyzer* class to calibrate the measured SAXS data. Furthermore, this class holds methods for calculating the lattice-plane ratios and determining the phase of the measured sample, returning an *LLCPhase* object for further analysis. *LLCPhase* is an abstract base class found in the *llcphase.py* module. It defines the interface of the different LLC phase objects, *HexagonalPhase*, *CubicPhase*, *LamellarPhase* and *IndeterminatePhase*, which are implemented as subclasses of *LLCPhase* in the module *llcphases.py*. While all phases contain the respective method for calculating the lattice parameter, additional methods are implemented as needed for further analysis of the different phases. Besides containing the aforementioned enum *SAXSStandards*, *enums.py* holds the enums *LLCPhases* with exact phase information, *LLCSpace­­Groups* with relevant space groups and *LLC­MillerIndices* with the Miller indices corresponding to the space groups.

### 2.3. APIs

#### 2.3.1. pyAnIML

AnIML provides a generic format to describe the methodology and results from measurements and a framework for the description of workflows and data structures. However, the format currently lacks an API, which facilitates the integration of different applications into a seamless workflow. Therefore, the *pyAnIML* library was developed as an API to AnIML.

Using the data validation and type enforcement package *pydantic* (https://pypi.org/project/pydantic/), *pyAnIML* imple­ments data models as Python objects rather than in a format-specific schema. As a consequence, the abstract object model is not restricted to a specific format, but enables the export of data and metadata to any XML or JSON format, including AnIML. *pyAnIML* implements the general structure of an AnIML document as Python class definitions. The objects are constructed as a tree of object relations. For example, the root-level structure of an AnIML document consists of *SampleSet*, *ExperimentStepSet* and *AuditTrailSet* class definitions which can be filled with data using dedicated *add* methods. These methods act as gateways to support the controlled addition of data to the AnIML container object, which is independent of native Python functions. The *Sample* elements within *SampleSet* are equipped with a unique and unambiguous *sampleID*, as well as a more human-readable *name* describing the samples’ content. These *Sample* elements are also referenced in the *Infrastructure* element of the various *ExperimentSteps* contained in the *ExperimentStepSet*. Each *ExperimentStep* represents a single SAXS measurement and is divided into the elements *Infrastructure*, *Method* and *Result*. The *Method* element contains information about the instrument used, the authors involved in that *ExperimentStep*, the software utilized and all the instrument parameters from the respective experiment. The data produced by an experiment are found in the *Result* element. Within this there is the *SeriesSet*, which contains one or more *Series* of data points, equipped with the *IndividualValueSet* and a unique *seriesID*, as well as further information about the data type (*seriesType*), the *dependency* of the data, its *plotScale* and, optionally, information about its *Unit*. As an alternative to *SeriesSet* elements, *Category* elements may contain one or more independent *Parameter* elements, also optionally equipped with information about the *Unit*. A *Unit* element has both a *label*, which is a string representation of the particular unit, and the *quantity* this unit belongs to. The *Unit* element may also contain any number of *SIUnit* elements, which represent the combination of basic SI units needed to represent the given unit by holding the symbol of an SI unit and its *factor*, *exponent* and *offset*.

All classes defined in *pyAnIML* include validated type annotations and *field* objects. The latter can be used to attach metadata to an attribute, such as a description or alias used in exported formats. In addition, these fields specify the location of an attribute in an XML document. For instance, a field may be set as an *attribute* which will then be written inside an *element* found in an XML element. The *field* object can also be specified to write specified *elements* when a certain data type is encountered. An example is the *IndividualValueSet* class, where data types are mapped to certain elements, which is a requirement of the AnIML specification. For instance, if a value found in each set is an integer, the resulting elements will then be tagged as an <I> element, whereas a floating data type would result in an <F> element.

*pyAnIML* is available both on GitHub (https://github.com/FAIRChemistry/pyAnIML) and in the Python Package Index (https://pypi.org/project/pyAnIML).

#### 2.3.2. *easyDataverse* and *pyDaRUS*

Data and metadata were stored as a *COMBINE* archive containing the AnIML-formatted document and other relevant files on the data repository of the University of Stuttgart, DaRUS (https://darus.uni-stuttgart.de/), a local installation of the generic research data repository Dataverse. The metadata of a data set are given by a customized searchable metadata block. A metadata block consists of fields that are either generic or specific to an application. For instance, the *Process* metadata block found on DaRUS reports on generic workflows and individual steps and can be applied to any application that involves complex workflows. Thus, the modularity of Dataverse addresses the interdisciplinary nature of modern science. For this work, the metadata blocks *EngMeta* (https://doi.org/10.18419/darus-500) and *Process* (https://doi.org/10.18419/darus-508) were used alongside the general and obligatory *Citation* block to report metadata on the workflow and results.

In order to enable a seamless workflow, the novel *pyDaRUS* library was developed and used. This library was written using the previously developed generic *easyDataverse* library which, on the basis of given metadata blocks in a Dataverse, generates an object model that can be used in conjunction with native I/O methods found in *easyDataverse.* The output of *easyDataverse* is based on the respective metadata configuration files of the Dataverse installation for which code is to be generated. APIs generated by *easyDataverse*, such as *pyDaRUS*, follow an object-oriented programming paradigm in which metadata blocks consist of attributes and subsequent objects, to which source fields found in the API are mapped. Once done, a data set is uploaded to the Dataverse installation for further editing or publication. A detailed description of how to generate an API for a given Dataverse installation can be found on the *easyDataverse* project page (https://github.com/gdcc/easyDataverse). Details on the creation of data sets for the University of Stuttgart Dataverse installation using *pyDaRUS* are also provided on GitHub (https://github.com/JR-1991/pyDaRUS).

### 2.4. SAS-workflows

**Figure 2:**
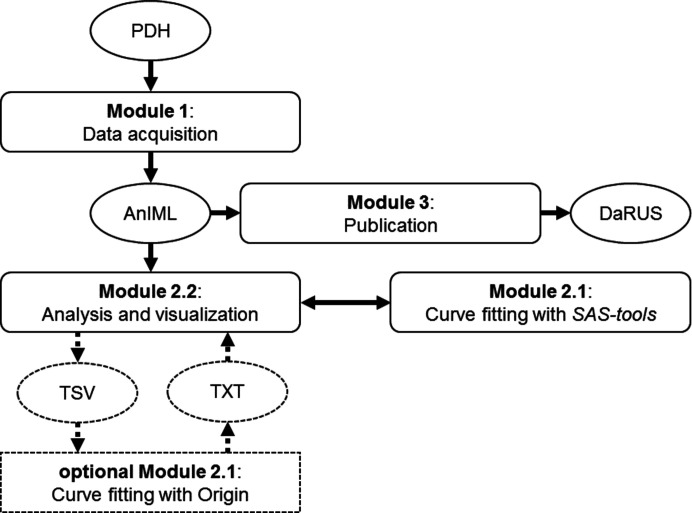
Flow diagram of the data management workflow for SAXS data, consisting of the three major modules (boxes) and the data exchange formats (ovals), with data flow indicated as arrows and optional parts as dotted lines.

To ensure maximum flexibility and adaptability of the RDM platform, the workflow for this work, contained in *SAS-workflows* (https://github.com/FAIRChemistry/SAS-workflows), was divided into three stand-alone modules (Fig. 2 ▸), each with one or more dedicated *Jupyter Notebooks*. Each of the *Notebooks* can be used as is, while still enabling extensibility.

#### 2.4.1. Module 1: Data acquisition

Naturally, different SAXS instruments output data in different file formats. For this work, the Anton Parr SAXSess mc^2^ was utilized and the *PDHReader* described in Section 2.2.1 was used. From these files, data and metadata were automatically parsed and read into the *Jupyter Notebook* (M1_AnIML.ipynb) and converted to AnIML using the *pyAnIML* API. The obtained AnIML document contains an entry for every sample measured. Additionally, the *Result* element is divided into a *Category* element for the actual measurement data of an experiment and the results of an analysis, in order to differentiate between measurements and results from data analysis. All relevant elements and attributes found in the raw data sets were mapped to the corresponding fields and can thus be used for further applications in the field of SAXS, beyond the scope of this work. Detailed information on the mapping from PDH to AnIML can be found in Table S2.

#### 2.4.2. Module 2: Curve fitting, analysis and visualization

**Submodule 2.1: Curve fitting**

Two alternative *Notebooks* are provided for curve fitting, a *Notebook* using the novel *curvefitting* module contained in the *analyzer* package of *SAS-tools* (M2-1_Curve_fitting.ipynb) and a *Notebook* for transferring data to *Origin* (M2-1_Origin.ipynb).

When using the internal *curvefitting* module, raw data are provided as *pandas.DataFrame*. After initialization of the *CurveFitting* class, the raw data are searched for peaks and the search result is plotted. The user then has the choice of setting the parameters for the fitting process manually or automatically and initializing the fitting process. The resulting fit is plotted and the determined positions of the peaks of interest are stored in a TXT file.

Alternatively, the data sets are converted to tab-separated values (TSV) format and transferred to *Origin*. In *Origin*, the Lorentzian fit available from *Origin Basic Functions* was used to fit reflections in recorded diffractograms with the aid of the Levenberg–Marquardt iteration algorithm according to the function

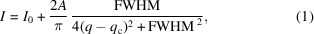

and the offset *I*_0_, peak center positions *q*_c_, peak width at half-maximum FWHM and area *A* were determined for each peak. Finally, the obtained fitting data (*I*_0_, *q*_c_, FWHM and *A* for each peak) were exported from *Origin* in plain TXT format (Fig. S1) into Submodule 2.2.

**Submodule 2.2: Data analysis and visualization**

Both the core calculations of the data analysis and the data visualization were performed in *Jupyter* using Python scripts. All results were appended to the same AnIML document created by module 1.

In the first *Notebook* (M2-2_Analysis.ipynb), the pandas.DataFrame containing the results of the Lorentzian fit from either the *SAS-tools*- or *Origin*-based curve fitting was used to calculate characteristic parameters. Using the measured and literature-known (Dorset, 1987 ▸) peak centers *q*_c_ from the calibration measurement, the respective measured *q*_c_ were corrected in a first step with the help of the *Prepare­Standard* class from *SAS-tools*. Moving to the *LLCAnalyzer*, the lattice-plane distance *d* was obtained using *d* = 2π/*q*_c_ and the ratio of the different *d* values was used to infer the LLC phase, returning the corresponding *LLCPhase* object. Additionally, the lattice parameters were calculated from the lattice-plane spacing and the corresponding Miller indices using the *LLCPhase*. The results from these calculations and all the metadata that are required for reproducibility were added to the AnIML document with the *pyAnIML* API. In addition, these results can be exported to a TSV-formatted plain text file, for convenient use in various other applications.

With the M2-2_Dif fractograms.ipynb *Notebook*, data are visualized using different file formats as input, such as the AnIML file or raw measurement data in PDH format. The *Jupyter Notebooks* allow for the creation of 2D diffractograms with one or several plots, and of 3D graphs containing multiple diffractograms depending on the sample mass fraction or the temperature. All graphs can be manipulated to meet requirements, including axes, scaling, figure size and resolution.

By manually adding the observed phase transitions to M2-2_Phase_diagrams.ipynb, a skeleton phase diagram can be plotted containing phase transition points, but not phase boundaries as these require basic knowledge of the phase behavior as well as experimental experience.

#### 2.4.3. Module 3: Publication

The Dataverse installation at the University of Stuttgart, DaRUS, was used for archiving and publication of data sets related to this work, providing a structured, searchable and public repository for unambiguous identification of data sets through a DOI. All SAXS data sets were uploaded to the Dataverse collection ‘SFB 1333A4 – Gießelmann/Bruckner group Dataverse’ (Giess *et al.*, 2022 ▸). For each data set, a metadata block was generated, which enables a search for the most relevant metadata (Table S3). The generation of the metadata block and the uploading of the data set were integrated into the *Jupyter Notebook* using the Python packages *pyDataverse* (https://pypi.org/project/pyDataverse/), *easyDataverse* (https://github.com/gdcc/easyDataverse) and *pyDaRUS* (https://github.com/JR-1991/pyDaRUS/). Thus, FAIR and scalable data management was ensured for the whole data lifecycle, from data acquisition to publication.

**Figure 3:**
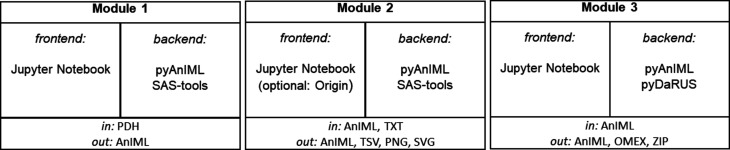
The structures of the three modules, detailing the frontend with which the user interacts, the backend which provides the functionality, and the input and output formats.

An AnIML document reports all relevant data and metadata of the experiment and provides a human-readable and machine-actable description of the experiment. However, it is also desirable to store the original data files, as well as complementary files such as additional descriptions of the experiment in plain text, log files, images or machine settings. Therefore, the AnIML document and the additional files were stored in a *COMBINE* archive (Fig. 3 ▸) using the Open Modeling Exchange (OMEX) format (Bergmann *et al.*, 2014 ▸). The *COMBINE* archive for SAXS data contains a mandatory file named manifest.xml, which lists the contents of the whole archive, one metadata.rdf file for each unique file contained in the archive which lists information about that file, an AnIML document as master file and any additional files. The package *python-libcombine* (https://pypi.org/project/python-libcombine/) was used for writing and reading the OMEX-formatted archive. The file extension ‘.zip’ was used instead of the customary ‘.omex’ suffix. While still formally complying with the OMEX standard, this file extension was chosen to show that the contents of such an archive are not necessarily connected to computational bio­sciences.

## 3. Use case: phase diagrams of aqueous octyltrimethylammonium bromide and chloride

To demonstrate the feasibility and usefulness of the SAS toolbox, it was applied to investigate LLC phases and phase transitions of aqueous octyltrimethylammonium bromide and chloride (C_8_TAB and C_8_TAC, respectively) by SAXS measurements.

### 3.1. Experimental methods

#### 3.1.1. Sample preparation

For the two surfactants octyltrimethylammonium chloride (O_8_TAC, Sigma Aldrich, ≥97%) and octyltrimethylammonium bromide (O_8_TAB, Sigma Aldrich, ≥98%), one sample series each, with increasing mass fractions of bi-distilled (bd) water, was prepared in 3 ml glass vials. The components were weighed with a Mettler Toledo scale (accuracy ±0.1 mg) and homogenized for 72 h on a roller (IKA, Roller 6 digital; 23.8°C, 5 r min^−1^) for surfactant mass fractions up to 50 wt%. Samples with higher mass fractions were mixed with the motor handpiece MHX/E from Xenox until homogeneous and left for 72 h in a ThermoShaker (biosan, PST-60HL; 40°C, 700 r min^−1^) for further equilibration. The composition of the samples was characterized by the total surfactant mass fraction *w*,

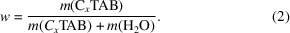




#### 3.1.2. SAXS scattering measurements

For SAXS measurements, glass No. 14 capillaries with a diameter of 0.7 or 0.9 mm from Hilgenberg were used. The marked tubes were filled with sample and fused with a lighter. All measurements were performed with a SAXSess mc^2^ diffractometer from Anton Paar, equipped with a TCS120 sample holder and a 1D CMOS detector from Dectris Mythen. With an ID3003 X-ray generator from Seifert (40 kV, 40 mA), Cu *K*α radiation was produced and line-collimated by the SAXSess mc^2^ system. The distance between the sample and the detector was calibrated with cholesteryl palmitate. Measurements were executed and monitored via the *SAXSquant* software (Anton Paar) and averaged over 60 individual measurements. Subsequently, the measured scattering curves were background corrected and deconvoluted using the same software in order to separate the scattering by the material from the beam profile.

### 3.2. Results

**Figure 4:**
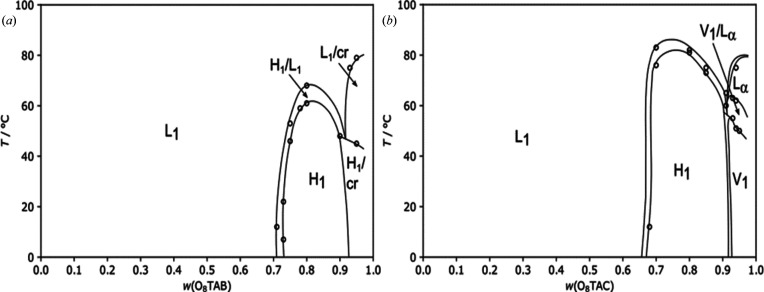
Phase diagrams for (*a*) the binary water/C_8_TAB system and (*b*) the water/C_8_TAC system as a function of the temperature *T* and the surfactant mass fraction *w*.

**Figure 5:**
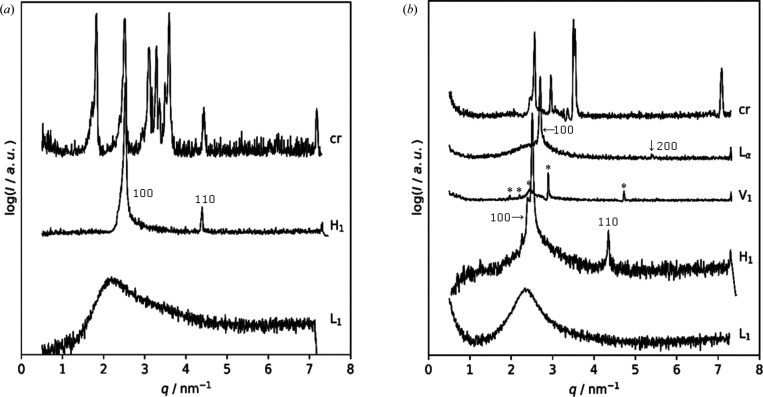
(*a*) SAXS scattering patterns obtained for crystalline (cr) C_8_TAB and observed phases of the C_8_TAB/water system (L_1_, H_1_). (*b*) SAXS patterns obtained for crystalline (cr) C_8_TAC and observed phases of the C_8_TAC/water system (L_1_, H_1_, V_1_, L_α_). The Miller indices for the cubic V_1_ peaks (indicated by asterisks, ‘*’) are 211, 220, 310, 321 and 611 with increasing scattering vector magnitude *q*.

**Figure 6:**
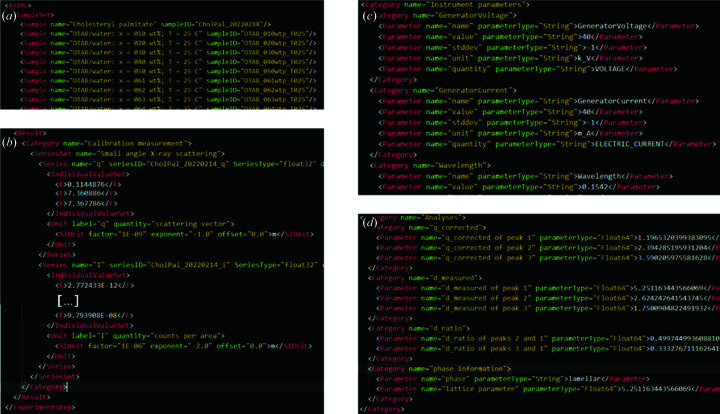
Excerpts from the AnIML file. (*a*) SampleSet, an overview of all samples found in the AnIML file, (*b*) measurement data obtained from the calibration measurement, (*c*) instrument parameters (generator voltage and current) and (*d*) analysis results, peak centers (q_corrected), lattice-plane distances (d_measured), ratios (d_ratio), LLC phase and lattice parameter.

**Figure 7:**
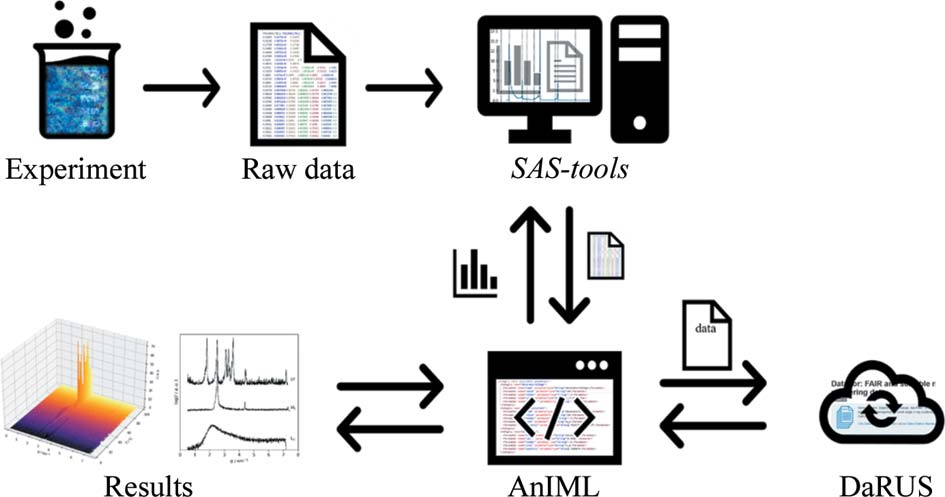
A diagram of the data management and analysis workflow. Experimental data are converted to an AnIML document. Analyses are carried out by reading data and writing results to the AnIML document. The AnIML document containing raw data and analysis results is uploaded to DaRUS and obtains a DOI, which makes it findable. The AnIML file is accessible and interoperable for both human and machine. With all experimental details contained in the AnIML file, measurements are reproducible and reusable.

With the aid of our RDM toolbox, collected data were converted to an AnIML file (module 1), analysis was performed (module 2) and all data uploaded to DaRUS (module 3). To a newly created AnIML file from the *Jupyter Notebook* M1, the recorded data from SAXS measurements were appended sample by sample. Each entry holds information about the sample, its purpose (calibration or sample data) and the measurement data themselves, as well as the author(s) and required device parameters. LLC phases are characterized by their characteristic lattice-plane ratios. Therefore, the observed peaks are fitted using a Lorentzian model. With module 2.1 this is possible both with external software and using the capabilities of the *SAS-tools* library presented here. Example results obtained from *Origin* in plain TXT format and fitting results from the *curvefitting-tool* are shown in Fig. 1 ▸. With the peak centers determined, module 2.2 was utilized to calculate the lattice-plane ratios to enable further determination of the corresponding LLC phase and calculation of the particular lattice parameters. The results of our investigations are summarized in two phase diagrams (Fig. 4 ▸) which were created using the skeleton diagrams from the *Notebooks* and filling in phase boundaries compliant with the physical restraints of phase behavior. Representative scattering curves of the observed individual LLC phases are shown in Fig. 5 ▸. Fig. 6 ▸ shows excerpts from the created AnIML file which give a short overview of the process of data conversion and analysis results. Fig. 7 ▸ shows a flow diagram of the complete workflow from experiment to the published DaRUS data set.

For the C_8_TAB/water system [Fig. 4 ▸(*a*)], we find a broad isotropic micellar solution L_1_ up to 73 wt% and a hexagonal H_1_ phase between 74 and 87 wt% of surfactant, which is stable up to 60°C. In this concentration range the hexagonal cell parameter decreases from *a* = 2.87 nm to *a* = 2.73 nm at 25°C. A narrow biphasic region separates the L_1_ and H_1_ phases from each other. Increasing the C_8_TAB mass fraction further leads to the formation of more extended biphasic regions, either between a crystalline (Cr) and the H_1_ phase or between a crystalline and the L_1_ phase. We did not inquire further on the structure of the crystalline phase.

The phase diagram of the C_8_TAC/water system is depicted in Fig. 4 ▸(*b*). Similar to the system with bromide, a broad H_1_ phase forms, which ranges from 68 to 93 wt% and is stable up to 81°C. The hexagonal cell parameter at 25°C decreases from *a* = 2.85 nm to *a* = 2.67 nm with increasing C_8_TAC mass fraction. Next to this phase, we identified two further LLC phases at very high surfactant mass fractions. The first one is a bicontinuous cubic V_1_ phase, which occurs between 94 and 97 wt% below 60°C. The relative position of the scattering peaks [Fig. 5 ▸(*b*)] reveals that this cubic phase is body centered and belongs to the space group 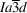 (Fig. S2). The cubic cell parameter for the 94 wt% sample at 50°C was calculated to be *a* = 2.17 nm. The second phase is the lamellar L_α_ phase, which appears at temperatures up to 70°C and is separated from the V_1_ phase by a biphasic region. A layer spacing of *d* = 2.28 nm was found for the L_α_ phase at 94 wt% and 64°C. At surfactant mass fractions of 97 wt% and above, biphasic regions with the crystalline phase occur.

The phase diagram of the C_8_TAB/water system [Fig. 4 ▸(*a*)] is in good agreement with the low-temperature phase diagram of the system reported by Fukada *et al.* (1998 ▸), which was recorded up to a maximum temperature of 20°C. When comparing the phase diagram of the C_8_TAB/water system and the phase diagrams of the C*_x_* TAB/water systems (*x* = 10, 12, 14, 16) (Varade *et al.*, 2008 ▸), we note that the phase transition between the L_1_ and H_1_ phases is shifted to significantly higher surfactant mass fractions and the clearing point is shifted to lower temperatures. C_8_TAB thus follows the trend of shifting phase transitions already observed when reducing the alkyl chain length from 16 to ten carbon atoms. This trend may be rationalized by the observation that specific phase transitions within LLC systems occur at specific volume fractions of the micelles (Mitchell *et al.*, 1983 ▸). While the correlation between the mass fraction of a surfactant and its volume fraction in water is not trivial, it is obvious that the total number of micelles must be larger for small micelles, as formed by surfactants with short alkyl chains, than for large micelles, to reach the same total volume fraction. Varade *et al.* (2008 ▸) calculated both the volume fraction and the radius of the cylindrical micelle of the lipophilic part of the surfactant in the H_1_ phase, showing that the values decrease from 51.4 to 43.3 vol.% and 1.87 to 1.30 nm, respectively, when going from the C_16_TAB to the C_10_TAB/water system. Unfortunately, a direct comparison with our system is not possible, because a fixed surfactant mass fraction of 65 wt% and a temperature of 40°C were chosen, at which the C_8_TAB/water system does not form an H_1_ phase. It would have been interesting to compare all five C*_x_* TAB/water systems which form an H_1_ phase under the same conditions (75 wt% and 40°C). However, the published SAXS raw data are not available in a reusable format, thus preventing a re-analysis and comprehensive comparison of the different systems.

For the lattice parameter of the H_1_ phase, data are available at roughly the same mass fraction (72–74 wt%) and temperature (25–27°C) for the C_10_TAB/water (Fukada *et al.*, 1998 ▸) and C_12_TAB/water (McGrath, 1995 ▸) systems. Gradually increasing the alkyl chain length leads to an increase in the lattice parameter from 2.87 nm in the C_8_TAB/water system to 3.42 nm and finally 3.91 nm. Thus, an increase in the hexagonal lattice parameter of roughly 0.5 nm can be deduced when increasing the alkyl chain length by two carbon atoms. Of course, the conclusion would be more substantial if more comparable data points were available.

Comparing the phase diagrams of the C_8_TAB/water system and the C_8_TAC/water system, we found two additional LLC phases, the V_1_ and the L_α_ phases, and a higher stability of the H_1_ phase in the concentration and temperature regime. This observation can be attributed to the effect known as the Hofmeister series, which classifies ions into structure-forming (kosmotropic) and structure-breaking (chaotropic) ions (Kang *et al.*, 2020 ▸). In the Hofmeister series, the chloride anion is centered in between the two extremes, while the bromide anion leans towards the chaotropic side, explaining the destabilization of the LLC phases in the C_8_TAB/water system. Our finding is supported by several studies which identified similarly pronounced counterion effects following the Hofmeister series for alkyltrimethylammonium surfactants (Blackmore & Tiddy, 1988*b* ▸; Liu & Warr, 2014 ▸).

## 4. Conclusion and outlook

The RDM toolbox presented here enables efficient handling of SAXS data. It provides the primary and derived data, as well as the experimental metadata, in the standardized exchange format AnIML, which enables future re-analysis. The workflow is immediately applicable using the popular *Jupyter Notebook* and Python packages, which can be installed on a wide range of hardware and operating systems. Its implementation is simple and transferable to other research groups. By developing dedicated *Jupyter Notebooks* for the individual steps of data collection, analysis and publication, the system is highly modular and therefore easily extensible, either by extending the *Jupyter Notebook* or by integrating other software such as *SigmaPlot* (Systat Software Inc.) or MATLAB.

The analysis of experimental data is not limited to managing single data sets. *SAS-tools* also provides a scalable toolbox for handling and analyzing large numbers of data sets. It facilitates analysis of the data by automated calculation of layer spacings and lattice parameters from the fitted data, and simplifies the publication of data with the generation of high-quality figures. Using AnIML, the seamless flow of data from acquisition to analysis and upload to the Dataverse repository removes the laborious and error-prone tasks of manual reformatting and editing of data and thus makes it more likely that researchers will make their data available.

The data management infrastructure fulfills the FAIR data principles. Our implementation of the Dataverse platform enables **findability** of data sets and public **access** to data sets. By using AnIML, the results of SAXS experiments and the derived structural properties become **interoperable** and can be **reused** for re-analysis. With the help of automated RDM tools, such as the toolbox presented in this publication, the rapidly growing amount of research data will be manageable in the future. Access to FAIR and scalable primary data reduces the need for repetition of experiments and is indispensable for high research quality.

The *SAS-tools* Python library was conceived and structured to be easily extensible. The focus on modularity and good programming practices provides convenient ways and the necessary platform to add other experimental methods and new tools to the software. As the name *SAS-tools* suggests, development efforts are being made to build a comprehensive Python library for small-angle scattering techniques. Methods such as 2D SAXS, tools for the analysis of thermotropic liquid crystals or data from SANS, and support for other instruments like the Bruker Nanostar are currently in progress.

## Supplementary Material

Additional details. DOI: 10.1107/S1600576723001577/jl5054sup1.pdf
