# Supplementary material for: FAIR and scalable management of small-angle X-ray scattering data
Source: J Appl Crystallogr. 2023 Mar 21;56(Pt 2):565–75. doi: 10.1107/S1600576723001577 (PMC10077856; doi:10.1107/S1600576723001577)
Supplement: Supplementary file 1 [file j-56-00565-sup1.pdf]

JOURNAL OF
APPLIED
CRYSTALLOGRAPHY

Volume 56 (2023)

Supporting information for article:

FAIR and scalable management of small-angle X-ray scattering data

Torsten Giess, Selina Itzigehl, Jan Range, Richard Schömig, Johanna R. Bruckner and Jürgen Pleiss

1. Supporting tables

Python package requirements: conda environment.yml files

The popular scientific computing package and environment manager *conda* was used in this work through *Miniconda3*, mainly for managing virtual environments. Only Python and Jupyter-related packages were installed with *conda*, all other packages and libraries were installed using *pip*. The "minimal" environment files only contain explicitly installed packages with their respective version for platform-agnostic environment creation. The "macos" files contain explicitly installed packages and all their dependencies with versions and exact build, the latter being MacOS/ARM64-specific, however.

Table S1. Environments used for this work

Environment type	Environment name	File name
base	base	base_minimal.yaml<br>base_macos.yaml
production	fairsaxs	fairsaxs_minimal.yaml<br>fairsaxs_macos.yaml

Mapping of PDH to AnIML

From the XML metadata footer of the PDH files, only the *column* and *parameter* elements as well as their children are currently mapped to AnIML, as these nodes contain the most essential information (**Table S2**).

Table S2. Mapping of PDH *column*, *parameter*, and *value* elements to AnIML *Series*, *Unit*, *Category* and *Parameter* elements.

**PDH**	**AnIML**
<column key=”…”>	<Series name=”…” seriesID=”…” SeriesType=”…” …>
<value key=”**unit**”>…</value>	<Unit label=”…” quantity=”**quantity**”>
<value key=”**quantity**”>…</value>	<SIUnit factor=”…” exponent=”…” offset=”…”><br>…</SIUnit><br></Unit>
</column>	</Series>
<parameter key=”…”>	<Category name=”…”>
<value key=”**name**”>…</value>	<Parameter name=”**name**” parameterType=”String”><br>…</Parameter>
<value key=”**value**”>…</value>	<Parameter name=”**value**” parameterType=”Float32”><br>…</Parameter>
<value key=”**stddev**”>…</value>	<Parameter name=”**stddev**” parameterType=”Float32”><br>…</Parameter>
<value key=”**unit**”>…</value>	<Parameter name=”**unit**” parameterType=”String”><br>…</Parameter>
<value key=”**quantity**”>…</value>	<Parameter name=”**quantity**” parameterType=”String”><br>…</Parameter>
</parameter>	</Category>

Structure of the DaRUS metadata blocks

On DaRUS, selected fields from the *Citation Metadata*, *Process Metadata*, and *Engineering Metadata* blocks were used in this work (**Table S3**).

Table S3. Implemented DaRUS metadata blocks and their fields used in this work.

Metadata block	Fields used
Citation Metadata	Title
	Author
	Contact
	Description
	Subject
	Keyword
	Topic Classification
	Grant Information
	Project
Process Metadata	Processing Methods
	Method Parameters
	Software
	Instruments
Engineering Metadata	Data Generation
	Measured Variables
	Controlled Variables

2. Supporting figures

Output of fit parameters from Origin

The TXT-formatted output from Origin provides information on the parameters of the Lorentzian fits on SAXS peaks (**Fig. S1**). The first column contains the intensity *I* as the dependent variable. The second column gives the parameter names followed by the fitted value and its standard deviation. The *t*-value is the ratio of the fitted value and its standard deviation. The *Prob*>|*t*|-value is the probability of the *t*-test and therefore allows inference to the significance of each parameter. Lastly, the dependency which is computed from the variance-covariance matrix further indicates the significance of each parameter. In the analysis and visualization toolkit merely the peak center values x_c were used for further calculations.

		value	Std dev	t-value	prob.>|t|	dependency
I	y0	0.00964	0.00958	1.00693	0.3475	0.97174
I	xc	1.96602	0.00212	927.72171	4.46519E-19	1.56017E-4
I	w	0.03667	0.01759	2.08479	0.07555	0.94112
I	A	0.00184	0.00128	1.43333	0.19488	0.98618

Figure S1. Section from exemplary TXT file with fitting data obtained from Lorentzian fit in Origin.

Indexation of the cubic LLC phase

The multiple scattering maxima of the cubic LLC phase may be assigned to various Miller' indices (*hkl*) resulting in different possible space groups. The best fit was obtained for the body-centered space group $Ia\bar{3}d$, as shown in **Fig. S2**. The linear regression between the measured values of d^{-1} and the theoretically calculated values of the assigned Miller's indices reveals an excellent agreement with $R^2 = 0.9997$.

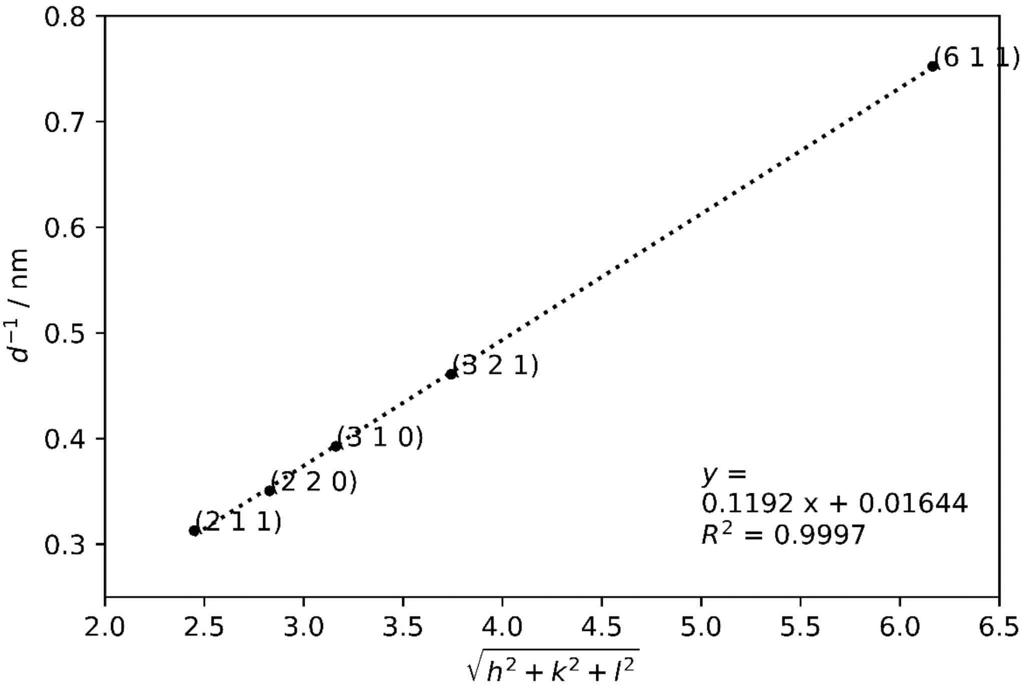

Figure S2. Plot of reciprocal lattice plane distance d^{-1} vs. square root of the sum of quadratic Miller's indices (*hkl*) for confirmation of the $Ia\bar{3}d$ space group of the cubic LLC phase.

3. Guide to the Notebooks

Guide to the Notebooks

Module 1: PDH to AnIML converter

Following the preparational steps and creation of an AnIML object, available PDH files for conversion are called via a respective directory (red box).

```
[8]: pdh_dir = PDHReader(path_to_datasets / "raw/OTAC_measurement_data/OTAC_000wtp_T025")
     dict_of_files = pdh_dir.available_files()
     for index, file in dict_of_files.items():
         print(f"{index}: {file}")

     17:08:33 - modules.pdhreader - DEBUG: Constructor called, 'PDHReader'@0x1c6b9e1af20 initialised.
     0: OTAC_001wtp_T025[5]
     1: OTAC_005wtp_T025[5]
     2: OTAC_010wtp_T025[5]
     3: OTAC_020wtp_T025[5]
     4: OTAC_030wtp_T025[5]
     5: OTAC_040wtp_T025[5]
     6: OTAC_050wtp_T025[5]
     7: OTAC_060wtp_T025[5]
     8: OTAC_061wtp_T025[5]
     9: OTAC_062wtp_T025[5]
     10: OTAC_063wtp_T025[5]
     11: OTAC_064wtp_T025[5]
     12: OTAC_065wtp_T025[5]
     13: OTAC_066wtp_T025[5]
     14: OTAC_067wtp_T025[5]
     15: OTAC_068wtp_T025[5]
     16: OTAC_069wtp_T025[5]
     17: OTAC_070wtp_T025[5]
     18: OTAC_080wtp_T025[5]
     19: OTAC_090wtp_T025[5]
     20: OTAC_091wtp_T025[5]
     [illegible]
```

As additional automation is indeed possible, the specification to a particular case as well as susceptability to error increases dramatically. Therefore, the AnIML object is built one dataset at the time. In the next step, one of the files from the previously printed `dict_of_files` is chosen by its index to proceed with.

3. Select the desired file either by name or by list index and extract the data as pandas dataframe:

```
[9]: pdh_file = dict_of_files[5]
     raw_dataframe = pdh_dir.extract_data(pdh_file)
     raw_metadata = pdh_dir.extract_metadata(pdh_file)
     print(raw_dataframe)

     17:09:29 - modules.pdhreader - DEBUG: Data extracted from 'OTAC_040wtp_T025[5]'.
     17:09:29 - modules.pdhreader - DEBUG: Metadata extracted from 'OTAC_040wtp_T025[5]'.
     17:09:29 - modules.pdhreader - DEBUG: Metadata casted to 'etree.ElementTree'.
           scattering_vector  counts_per_area
     0              0.114488     3.473180e-16
     1              0.121128     4.665860e-09
     2              0.127769     2.202300e-08
     3              0.134409     1.146515e-09
     4              0.141050     1.624531e-09
     ...                 ...              ...
     1048           7.431255     6.507863e-04
     1049           7.437650     2.467263e-04
     1050           7.444044     6.541748e-04
     1051           7.450438     1.549244e-05
     1052           7.456831     1.350767e-05

     [1053 rows x 2 columns]
```

With the data at hand, the elements of the AnIML object are built up from bottom to top. Firstly, the experiment and sample are labelled with name (`experiment_name`) and ID

(`Sample_id`). These names can be assigned to the respective variables as a string containing text as well as created from a file name (here `pdh_file`). This, however, requires consistent naming of all measurement files.

4. Start building up the AnIML document. Create a new sample with an ID and name and add it to the AnIML object:

```
[10]: experiment_name = f"{pdh_file[:4]}/water: x = {pdh_file[5:8]} wt%; T = {pdh_file[-5:-3]} C"
      print(experiment_name)

      OTAC/water: x = 040 wt%; T = 25 C

[11]: new_sample = Sample(
          id=pdh_file.replace("%", "p")[:-3],
          name=experiment_name
      )
      print(new_sample)

      Sample(name='OTAC/water: x = 040 wt%; T = 25 C', id='OTAC_040wtp_T025', properties=[])

[12]: animl_doc.add_sample(new_sample)
```

Next, the experiment step object is created by assigning as name and an ID, similarly to the previous step (Case a). Alternatively, an existing experiment step within an AnIML object can be chosen (Case b). Additionally, a sample reference is added to the `experiment_step` providing the sample object, its role and purpose (Step 6)

5. Create or access an experiment step for the AnIML object, providing it with a name and an ID:

- Case a) Create a new experiment step object:

```
[13]: experiment_step = ExperimentStep(
          name=f"Sample data for {experiment_name}",
          experiment_step_id=pdh_file.replace("%", "p")[:-3]
      )
      print(experiment_step)

      ExperimentStep(name='Sample data for OTAC/water: x = 040 wt%; T = 25 C', experiment_step_id='OTAC_040wtp_T025', inf
      rastructure=Infrastructure(sample_references=SampleReferenceSet(sample_references=[])), method=Method(methods=[]),
      result=Result(results=[]))
```

- Case b) Access an existing experiment step within an AnIML document:

```
[ ]: available_experiment_steps = experiment_step = animl_doc.experiment_step_set.experiment_steps
     print([step.name for step in available_experiment_steps])

[ ]: experiment_step = available_experiment_steps[0]
```

Step 7 offers the opportunity to add authors, device and software information to the AnIML object as `instrument_parameters`.

In the next step, actual measurement data is added as a series for every dimension. For that purpose, a `Category` is created or an existing one is accessed. The units of the columns are extracted from the metadata of the measurement files and the actual values are stored in `IndividualValueSets`. Another `SeriesSet` is created holding the measurement data and associated information which is added to the `Category`. The `Category`, in turn, is then added to the `experiment_step`. The experiment step which now contains all the information of one measurement is finally added to the AnIML object.

```
[24]: q_values = IndividualValueSet(
          raw_dataframe["scattering_vector"].tolist()
      )
      q = Series(
          name="q",
          id=f"{pdh_file.replace('%', 'p')[:-3]}_q",
          unit=q_unit,
          individual_value_set=q_values,
          data_type="float32",
          dependency="dependent",
          plot_scale="linear"
      )
      i_values = IndividualValueSet(
          raw_dataframe["counts_per_area"].tolist()
      )
      i = Series(
          name="I",
          id=f"{pdh_file.replace('%', 'p')[:-3]}_i",
          unit=i_unit,
          individual_value_set=i_values,
          data_type="float32",
          dependency="dependent",
          plot_scale="linear"
      )
```

9. Create one or more sets for series belonging together, provide the set with a name, add it to the category object, and add it to the experiment step object:

```
[29]: new_set = SeriesSet(
          name=f"Small angle X-ray scattering",
          series=[q, i]
      )
```

```
[30]: category.add_content(new_set)
```

```
[31]: experiment_step.add_result(category)
```

10. Finally, add the now fully built experiment step object to the AnIML object:

```
[32]: animl_doc.add_experiment_step(experiment_step)
```

In a last step, an XML-formatted string is created from the AnIML object and serialized to the given AnIML document.

To add further datasets to the AnIML document, an existing document is called (Step 1, Case b), following steps are carried out as before. The finished AnIML document now contains all information needed to recreate a similar experiment as well as raw data of the measurements. Exemplary excerpts are shown in the following.

In the beginning of the AnIML document an overview over all contained sample data is given. For each dataset the instrument information is given followed by the result which contains two series holding the scattering vector (in nm^{-1}) and corresponding intensity (in counts per area).

```
<AnIML>
  <SampleSet>
    <Sample name="Cholesteryl palmitate" sampleID="CholPal_20220214"/>
    <Sample name="OTAB/water: x = 010 wt%; T = 25 C" sampleID="OTAB_010wtp_T025"/>
    <Sample name="OTAB/water: x = 020 wt%; T = 25 C" sampleID="OTAB_020wtp_T025"/>
    <Sample name="OTAB/water: x = 030 wt%; T = 25 C" sampleID="OTAB_030wtp_T025"/>
    <Sample name="OTAB/water: x = 040 wt%; T = 25 C" sampleID="OTAB_040wtp_T025"/>
    <Sample name="OTAB/water: x = 050 wt%; T = 25 C" sampleID="OTAB_050wtp_T025"/>
    <Sample name="OTAB/water: x = 061 wt%; T = 25 C" sampleID="OTAB_061wtp_T025"/>
    <Sample name="OTAB/water: x = 062 wt%; T = 25 C" sampleID="OTAB_062wtp_T025"/>
    <Sample name="OTAB/water: x = 063 wt%; T = 25 C" sampleID="OTAB_063wtp_T025"/>
```

```xml
<Category name="GeneratorVoltage">
  <Parameter name="name" parameterType="String">GeneratorVoltage</Parameter>
  <Parameter name="value" parameterType="String">40</Parameter>
  <Parameter name="stddev" parameterType="String">-1</Parameter>
  <Parameter name="unit" parameterType="String">k_V</Parameter>
  <Parameter name="quantity" parameterType="String">VOLTAGE</Parameter>
</Category>
<Category name="GeneratorCurrent">
  <Parameter name="name" parameterType="String">GeneratorCurrent</Parameter>
  <Parameter name="value" parameterType="String">40</Parameter>
  <Parameter name="stddev" parameterType="String">-1</Parameter>
  <Parameter name="unit" parameterType="String">m_A</Parameter>
  <Parameter name="quantity" parameterType="String">ELECTRIC_CURRENT</Parameter>
</Category>
<Category name="Wavelength">
  <Parameter name="name" parameterType="String">Wavelength</Parameter>
  <Parameter name="value" parameterType="String">0.1542</Parameter>
  <Parameter name="stddev" parameterType="String">-1</Parameter>
  <Parameter name="unit" parameterType="String">n_m</Parameter>
  <Parameter name="quantity" parameterType="String">LENGTH</Parameter>
</Category>
<Category name="SampleDetector">
```



```xml
<Result>
  <Category name="Calibration measurement">
    <SeriesSet name="Small angle X-ray scattering">
      <Series name="q" seriesID="CholPal_20220214_q" SeriesType="float32" dependency="dependent" plotScale="linear">
        <IndividualValueSet>
          <F>0.1144876</F>
          <F>0.1211282</F>
          <F>0.1277688</F>
          <F>0.1344093</F>
          <F>0.1410499</F>
          <F>0.1476904</F>
          <F>0.154331</F>
          <F>0.1609715</F>
          <F>0.167612</F>
          <F>0.1742525</F>
          <F>0.180893</F>
          <F>0.1875335</F>
          <F>0.194174</F>
          <F>0.2008145</F>
          <F>0.2074549</F>
          <F>0.2140954</F>
          <F>0.2207358</F>
          <F>0.2273762</F>
```


Module 2: Analysis and visualization toolkit

Submodule 2.1: Lorentzian fit with Origin

Lorentzian fits of the measured peaks are carried out using an “external” software (Origin). For this purpose, the data stored in the AnIML document is converted to a TSV file.

OTAB_010wtp_T025_q	OTAB_010wtp_T025_i	OTAB_020wtp_T025_q	OTAB_020wtp_T025_i	OTAB_030wtp_T025_q	OTAB_			
0.1144876	5.022773e-05	0.1144876	0.0001425089	0.1144876	0.0001344119	0.1144876	0.0001398	
0.1211282	3.202732e-05	0.1211282	8.237501e-05	0.1211282	9.126113e-05	0.1211282	0.0001441	
0.1277688	4.007452e-05	0.1277688	3.089387e-05	0.1277688	7.567179e-05	0.1277688	8.261919e	
0.1344093	4.159042e-05	0.1344093	6.727684e-05	0.1344093	8.193601e-05	0.1344093	7.928888e	
0.1410499	2.097933e-05	0.1410499	3.717403e-05	0.1410499	2.555185e-05	0.1410499	7.838917e	
0.1476904	9.018931e-06	0.1476904	2.099846e-05	0.1476904	1.284285e-05	0.1476904	5.895369e	
0.154331	1.01313e-05	0.154331	8.511214e-06	0.154331	1.797177e-05	0.154331	7.842683e-06	
0.1609715	4.636202e-06	0.1609715	4.804273e-06	0.1609715	1.130173e-05	0.1609715	1.780386e	
0.167612	3.735314e-06	0.167612	3.235822e-06	0.167612	1.172821e-05	0.167612	6.047424e	
0.1742525	9.089967e-07	0.1742525	1.307362e-06	0.1742525	9.442724e-06	0.1742525	1.977883e	
0.180893	4.62742e-07	0.180893	1.00951e-06	0.180893	2.491483e-06	0.180893	1.644553e-07	0
0.1875335	6.560143e-07	0.1875335	4.86578e-06	0.1875335	2.916286e-06	0.1875335	2.274738e-06	
0.280499	2.158597e-05	0.280499	1.048111e-05	0.194174	4.545391e-08	0.2871393	3.978696e	
0.2871393	0.0009829878	0.2871393	0.0007430934	0.280499	7.802804e-06	0.2937796	0.0012929	
0.2937796	0.005795988	0.2937796	0.00216936	0.2871393	0.0002224351	0.3004198	0.003342162	0.293
0.3004198	0.008043355	0.3004198	0.005005319	0.2937796	0.001319709	0.3070601	0.001220094	0.3004198
0.3070601	0.008263934	0.3070601	0.005745596	0.3004198	0.005770913	0.3137003	0.003158536	0.3070601
0.3137003	0.007421182	0.3137003	0.003981604	0.3070601	0.005733076	0.3203405	0.008937276	0.3137003
0.3203405	0.01779094	0.3203405	0.01231899	0.3137003	0.002706087	0.3269806	0.004350444	0.3203405
0.3269806	0.009802807	0.3269806	0.006323036	0.3203405	0.01359287	0.3336208	0.003994879	0.3269806
0.3336208	0.00896758	0.3336208	0.01062656	0.3269806	0.005441964	0.3402609	0.01207813	0.3336208
0.3402609	0.02386342	0.3402609	0.01754615	0.3336208	0.008931588	0.346901	0.01069388	0.3402609

Submodule 2.2: Analysis

In order to determine the lyotropic liquid crystalline (LLC) phase and the corresponding lattice parameter *a*, several steps are necessary. To be able to add the analysis data to the AnIML document afterwards, a respective `experiment_step` must be accessed and added a new `Category` which will hold the analyses. The next step involves the import of Lorentzian fit data obtained from Origin (or any other analysis software). The available files are stored in a data frame from which a file can be chosen by its list index later.

Firstly, the literature *q*-values (in nm^{-1}) are calculated from given *d*-values (given in Å). Measured *q*-values are corrected with the slope and intercept obtained from plotting literature versus measured *q*-values of the cholesteryl palmitate measurement (list index 0). The file to complete the analysis with is chosen via `dict_of_df[available_txt_files[`*list index*`].name]`.

Calculate the scattering vectors for calibration from literature lattice plane distances (given in Angstrom).

[ ]:
```
prepare_standard = PrepareStandard(SAXSStandards.CHOLESTERYL_PALMITATE)
q_cholpal_literature = prepare_standard.q_std_lit
print(q_cholpal_literature)
```

Calibrate the peak centers of a measurement (element from `available_txt_files`) with the calibration line. Then calculate the lattice plane ratio from `q_corrected`:

[ ]:
```
slope_and_intercept = prepare_standard.calculate_linear_regression(
    q_std_meas=dict_of_df[available_txt_files[0].name]["value"].tolist()
)
print(slope_and_intercept)
```

[ ]:
```
llc_analyzer = LLCAnalyzer()
llc_analyzer.calibrate_data(
    slope=slope_and_intercept[0],
    q_meas=dict_of_df[available_txt_files[0].name]["value"].tolist(),
    intercept=slope_and_intercept[1]
)
q_corrected = llc_analyzer.q_corr
print(q_corrected)
```

The calculated and corrected *q*-values of the scattering maxima are subsequently added to the AnIML document within a `Category`.

Add corrected q values to the AnIML document:

```
[20]: subcategory = Category(name="q_corrected")
```

```
[21]: for i, q in enumerate(q_corrected):
          new_parameter = Parameter(
              name=f"q_corrected of peak {i+1}",
              parameter_type=infer_type(q),
              value=q
          )
          subcategory.add_content(new_parameter)
```

```
[22]: new_category.add_content(subcategory)
```

Next, the lattice plane distance is calculated (in nm) from the corrected q-values and subsequently added to the AnIML document in another `Category`. The lattice plane ratio d, as well, is calculated and added to the AnIML document.

Calculate lattice plance distances d and their ratios from the corrected q values:

```
[ ]: llc_analyzer.calculate_lattice_ratio()
     d_measured = llc_analyzer.d_measured
     print(d_measured)
     d_ratio = llc_analyzer.d_ratio
     print(d_ratio)
```

With this information, the LLC phase can now be determined. As certain phases exhibit characteristic lattice plane ratios they are checked against given conditions. If a phase is determined, the corresponding lattice constant a is calculated accordingly. If the phase is indeterminate further analysis by visualization can be carried out (see **Submodule 3.2: Diffractograms**).

Determine the LLC phase from the lattice plane ratio and calculate the respective lattice parameter a:

```
[ ]: phase = llc_analyzer.determine_phase()
     print(phase)
```

```
[ ]: phase.calculate_lattice_parameters(d_meas=d_measured)
     phase_information = phase.phase_information
     print(phase_information)
```

If a cubic phase is interpreted from the diffractograms (or the above script) the space group can be specified by comparing measured reciprocal d^{-1}-values versus $\sqrt{h^2 + k^2 + l^2}$. The closer the R^2-value of the resulting plot is to unity; the more likely the assigned Miller indices and corresponding space group are. With another chapter in this notebook, the space group specifying the cubic phase can be determined by creating such a plot by input of different Miller indices. Obtained results can afterwards be added to the AnIML file.

Submodule 2.2: Diffractograms

In a first part of this Notebook, data visualization of two parameters is possible with data from the AnIML document. Therefore, a respective AnIML document is chosen by its directory, the measurement data of one or more samples selectable through their IDs in `files_to_plot`. With the data from `files_to_plot`, two-parameter plots are created for each dataset.

Import of data from AniML

Export q and I to TSV for plotting:

```
[45]: path_to_AnIML_file = path_to_datasets / f"processed/fairsaxs_220512.animl"
```

```
[46]: with path_to_AnIML_file.open("r") as f:
          xml_string = f.read()
          animl_doc = AnIMLDocument.fromXMLString(xml_string)
```

```
[47]: reader = SeriesReader(animl_doc)

      17:49:04 - modules.seriesreader - DEBUG: Constructor called, 'SeriesReader'@0x2410f122e30 initialised.
      17:49:04 - modules.seriesreader - DEBUG: Destructor called, 'SeriesReader'@0x2410ac238e0 deleted.
```

```
[48]: list_of_IDs = reader.available_seriesIDs()
      print(list_of_IDs)

      ['CholPal_20220214', 'OTAB_010wtp_T025', 'OTAB_020wtp_T025', 'OTAB_030wtp_T025', 'OTAB_040wtp_T025', 'OTAB_050wtp_T
      025', 'OTAB_061wtp_T025', 'OTAB_062wtp_T025', 'OTAB_063wtp_T025', 'OTAB_064wtp_T025', 'OTAB_065wtp_T025', 'OTAB_066
      [illegible]
```

```
[49]: files_to_plot = [file for index, file in enumerate(list_of_IDs) if file.startswith("OTAB")]
      print(files_to_plot)

      reader.add_seriesID(files_to_plot)
      dataframe = reader.create_dataframe()

      ['OTAB_010wtp_T025', 'OTAB_020wtp_T025', 'OTAB_030wtp_T025', 'OTAB_040wtp_T025', 'OTAB_050wtp_T025', 'OTAB_061wtp_T
      025', 'OTAB_062wtp_T025', 'OTAB_063wtp_T025', 'OTAB_064wtp_T025', 'OTAB_065wtp_T025', 'OTAB_066wtp_T025', 'OTAB_067
      wtp_T025', 'OTAB_068wtp_T025', 'OTAB_069wtp_T025', 'OTAB_070wtp_T025', 'OTAB_071wtp_T025', 'OTAB_073wtp_T025', 'OTA
      B_074wtp_T025', 'OTAB_075wtp_T025', 'OTAB_078wtp_T025', 'OTAB_079wtp_T025', 'OTAB_080wtp_T025', 'OTAB_090wtp_T025',
      'OTAB_100wtp_T025', 'OTAB_078wtp_T058', 'OTAB_078wtp_T060', 'OTAB_082wtp_T025', 'OTAB_093wtp_T025', 'OTAB_100wtp_T0
      95']
```

```
[50]: path_to_TSV_file = path_to_datasets / f"processed/fairsaxs_220512.tsv"
```

```
[51]: dataframe.to_csv(
          path_or_buf=path_to_TSV_file,
          sep="\t",
          index=False
      )
```

```
[52]: for index in range(0, (len(dataframe.columns)),2):
          data = pd.read_table(path_to_TSV_file,
                               usecols = [index, (index+1)],
                               names = ["q", "I"],
                               header = 1,
                               engine = "python"
                              )

          _ = data[data["q"] >= 0.5]
          plot_data = _[_["q"] <= 7]

          scattering_vector = plot_data["q"]
          counts_per_area = plot_data["I"]

          plt.plot(scattering_vector,
                   counts_per_area,
                   linestyle = "-",
                   marker = ",",
                   label = (list(dataframe.columns)[index])[0:-2],
                   color = "black")
          plt.xlim(0,7)
          plt.xlabel("$q$ / $\mathrm{nm}^{-1}$")
          plt.yscale("log")
          plt.ylabel("log($I$ / a.u.)")
          plt.legend(frameon=False)
          plt.show()
```

Two-parameter plots showing two or more diffractograms in one graph are possible, too. These plots are created similarly from the selected data in `files_to_plot`. Additionally, a phase description can be added to each dataset by specifying it in the list phase. This list should contain and equal number of entries as `files_to_plot`.
Furthermore, plots visualizing three parameters can be created from raw datasets. The files contained in a selected folder are shown as `meas_files`. Corresponding mass fractions are added to the list `mass_fractions`. A colormap is chosen according to the number (`n_meas`) of datasets. Each measurement is then added to the figure, as shown by the output of the cell. The figure can be created as is or adapted to best meet individual requirements (e.g. change axis label, ticks, scale, etc.)

For mass fration dependency, go to the measurement folder (`datasets/raw/`) containing the data for visualization and create a list of files:

```
[13]: path_to_T_series = path_to_datasets / "raw/OTAB_measurement_data/OTAB_000wtp_T025"
```

```
[14]: files = path_to_T_series.glob("*.pdh")
meas_files = [file for file in list(files) if file.is_file()]
meas_files.sort(reverse=True)

print([measurement.stem for measurement in meas_files])
```

```
['OTAB_100wtp_T025[5]', 'OTAB_090wtp_T025[5]', 'OTAB_080wtp_T025[5]', 'OTAB_079wtp_T025[5]', 'OTAB_078wtp_T025[5]', 'OTAB_075wtp_T025[5]', 'OTAB_074wtp_T025[5]', 'OTAB_073wtp_T025[5]', 'OTAB_071wtp_T025[5]', 'OTAB_070wtp_T025[5]', 'OTAB_069wtp_T025[5]', 'OTAB_068wtp_T025[5]', 'OTAB_067wtp_T025[5]', 'OTAB_066wtp_T025[5]', 'OTAB_065wtp_T025[5]', 'OTAB_064wtp_T025[5]', 'OTAB_063wtp_T025[5]', 'OTAB_062wtp_T025[5]', 'OTAB_061wtp_T025[5]', 'OTAB_060wtp_T025[5]', 'OTAB_050wtp_T025[5]', 'OTAB_040wtp_T025[5]', 'OTAB_030wtp_T025[5]', 'OTAB_020wtp_T025[5]', 'OTAB_010wtp_T025[5]']
```

Add the measured mass fractions to `mass_fractions` and instantiate the `inferno` colormap by storing the number of measurements in `n_meas`.

```
[15]: mass_fractions = [10, 20, 30, 40, 50, 60, 61, 62, 63, 64, 65, 66, 67, 68, 69, 70, 71, 73, 74, 75, 78, 79, 80, 90, 100]
mass_fractions.sort(reverse=True)
```

```
[16]: cmap = mpl.cm.gist_rainbow
n_meas = len(meas_files)
print(n_meas)
```

```
25
```

Instantiate the figure and add all measurements to it:

```
[17]: fig = plt.figure()
ax = plt.axes(projection="3d")
ax.figure.set_size_inches(10, 10)
ax.set_xlabel("$q$ / $\mathrm{nm}^{-1}$")
ax.xaxis.set_ticks([0, 1, 2, 3, 4, 5, 6, 7])
ax.set_ylabel("$x$ / wt%")
ax.set_ylim(0, 100)
ax.set_zlabel("$I$ / $a.u.$")

for measurement in range(len(mass_fractions)):
    print(f"Adding {meas_files[measurement].stem} to figure.")

    data = pd.read_table(
        meas_files[measurement],
        delimiter="   ",
        usecols=[0, 1],
        names=["q", "I"],
        header=5,
        [illegible]
    for data_points in range(len(scattering_vector)):
        mass_fraction.insert(data_points, mass_fractions[measurement])

    ax.plot(
        scattering_vector,
        mass_fraction,
        counts_per_area,
        linestyle="-",
        marker=",",
        color=cmap(measurement / float(n_meas)),
    )

plt.show()
```

```
Adding OTAB_100wtp_T025[5] to figure.
Adding OTAB_090wtp_T025[5] to figure.
Adding OTAB_080wtp_T025[5] to figure.
Adding OTAB_079wtp_T025[5] to figure.
Adding OTAB_078wtp_T025[5] to figure.
Adding OTAB_075wtp_T025[5] to figure.
Adding OTAB_074wtp_T025[5] to figure.
```

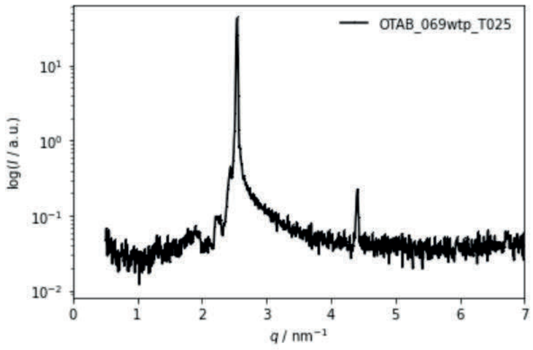

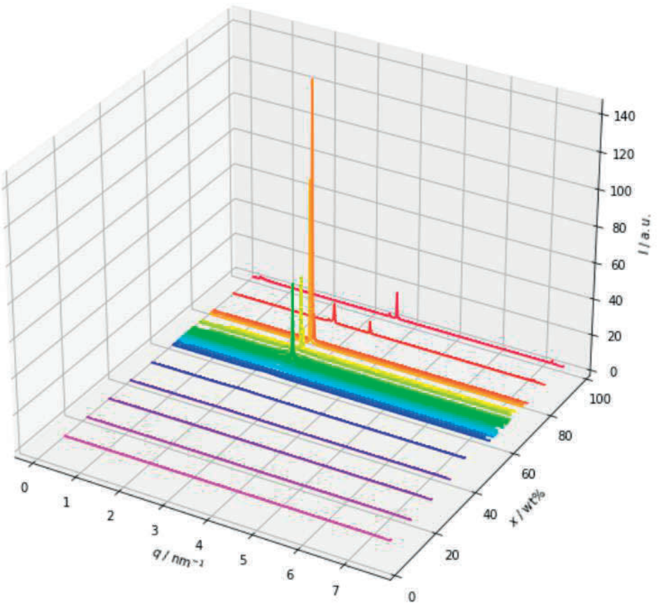

Similar graphs can be created for temperature dependency. The procedure is therefore similar to the previously described. A folder holding the desired measurement series is selected as well as the temperatures and colormap. The figure is instantiated and all graphs added consecutively.

Submodule 2.2: Phase diagrams

After all phase transitions are determined, they can be added to the lists `mass_fraction` and `temperature` to create a "skeleton" phase diagram.

Phasediagrams with Python

Define the name of your sample which will be added to the `x-axis` label. To `mass_fraction` add the mass fractions, to `temperature` the corresponding temperatures of phase transitions that were found while inspecting various diffractograms.

```
[ ]: name = "O$_8$TAB"
```

```
[ ]: mass_fraction = [0.71, 0.73, 0.73, 0.75, 0.75, 0.78, 0.80, 0.80, 0.90, 0.93, 0.95, 0.95, 0.95]
```

```
[ ]: temperature = [12, 7, 22, 46, 53, 59, 61, 68, 48, 75, 22, 45, 79]
```

Create the "skeleton plot":

```
[ ]: plt.plot(mass_fraction,
             temperature,
             marker='o',
             markersize=4,
             mfc="None",
             linestyle="None",
             color="black",
             )
     plt.xlabel(f"$w$({name})")
     plt.xlim(0, 1)
     plt.xticks(np.arange(0,1.1,step=0.1))
     plt.ylabel("$T$ / °C")
     plt. ylim(0, 100)
     plt.show
```

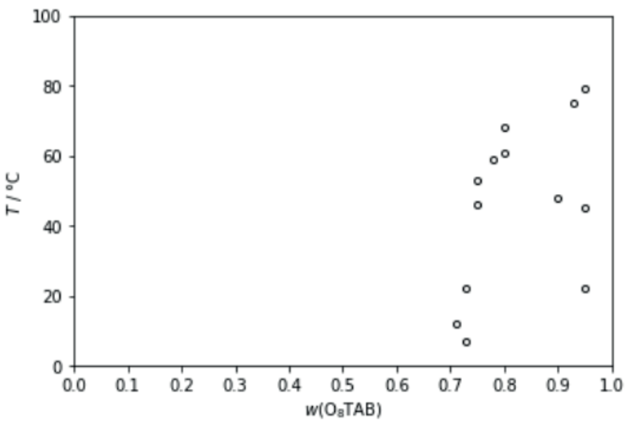

Module 3: OMEX format and dataverse handler

With this Notebook, the metadata block as well as an OMEX or ZIP archive is created to upload to DaRUS. First, after the preparational steps, a respective AnIML document is accessed and read, as well as necessary pyDaRUS objects created.

1. Give path to AnIML document to be uploaded to DaRUS in form of a pathlib Path:

```
[ ]: path_to_AnIML_file = path_to_datasets / f"processed/fairsaxs_220512.animl"
```

2. Read document as string and create AnIML object from it:

```
[ ]: with path_to_AnIML_file.open("r") as f:
         xml_string = f.read()
         animl_doc = AnIMLDocument.fromXMLString(xml_string)
```

3. Create the necessary pyDaRUS objects to be filled with metadata from the AnIML document. The title of the dataset is also provided here as an argument to the citation block:

```
[ ]: citation_block = Citation()
     process_block = Process()
     engineering_block = EngMeta()
```

In a next step, the citation block object is filled by adding various information including the title, authors, and keywords among others. This information is added manually and can therefore be manipulated accordingly.

4. Add general citation information to the citation block object that cannot be inferred from the AnIML document itself:

```
[ ]: citation_block.title = "FAIR and scalable management of small-angle X-ray scattering data"
```

```
[ ]: citation_block.add_author("Giess, Torsten", "University of Stuttgart", IdentifierScheme.orcid, "0000-0002-8512-8606")
     citation_block.add_author("Itzigehl, Selina", "University of Stuttgart", IdentifierScheme.orcid, "0000-0003-0311-5930")
     citation_block.add_author("Range, Jan", "University of Stuttgart", IdentifierScheme.orcid, "0000-0001-6478-1051")
     citation_block.add_author("Bruckner, Johanna R.", "University of Stuttgart", IdentifierScheme.orcid, "0000-0001-7183-6532")
     citation_block.add_author("Pleiss, Jürgen", "University of Stuttgart", IdentifierScheme.orcid, "0000-0003-1045-8202")
```

```
[ ]: citation_block.add_contact("Pleiss, Juergen", "University of Stuttgart", "juergen.pleiss@itb.uni-stuttgart.de")
```

```
[ ]: citation_block.add_description(f"This dataset contains the AnIML document, as well as all additonal files relevant to '{citation_bloc
```

```
[ ]: citation_block.subject = [SubjectEnum.chemistry, SubjectEnum.physics, SubjectEnum.computer_and__information__science]
```

```
[ ]: citation_block.add_keyword(
         term="AnIML",
         vocabulary="Wikidata",
         vocabulary_url="https://www.wikidata.org/wiki/Q97359795"
     )
     citation_block.add_keyword(
         term="Project Jupyter",
         vocabulary="Wikidata",
         vocabulary_url="https://www.wikidata.org/wiki/Q55630549"
     )
     citation_block.add_keyword(
         term="Surfactants",
```

For the process block object, information about the experiments are gathered from the AnIML document. The experiment, its corresponding parameters, units and methods are accessed and added to the block object. Furthermore, the process block object is filled with information about the measurements including the instrument and the software used to gather the data as well as the data itself.

6. Retrieve experiment parameters, remove duplicate entries, and transform the resulting list into a comma-separated string:

```
[ ]: for experiment_step in animl_doc.experiment_step_set.experiment_steps:
         for category in experiment_step.result.results:
             if category.name == "Analyses":
                 continue
             for series_set in category.content:
                 unique_parameters = {series.unit.label: series.unit.quantity for series in series_set.series}
```

```
[ ]: method_parameter_labels = ", ".join(unique_parameters.keys())
```

7. Add experiment name and its relevant parameters to the process metadata block object:

```
[ ]: process_block.add_processing_methods(
         name=method_name,
         parameters=method_parameter_labels,
     )
```

8. Create dictionaries of the relevant parameters' names and units:

```
[ ]: parameter_names = unique_parameters
     parameter_names["T"] = "temperature"
     parameter_names["w"] = "mass fraction"
```

```
[ ]: parameter_units = {
         "q": "nm^-1",
         "I": "a.u.",
         "T": "°C",
         "w": "wt%"
     }
```

9. Add the full parameter information to the process metadata block object:

```
[ ]: for parameter in unique_parameters.keys():
         process_block.add_method_parameters(
             name=parameter_names[parameter],
             symbol=parameter,
             unit=parameter_units[parameter]
         )
```

Lastly, the engineering block object is holding information about the variables. The DaRUS dataset object is then created by adding all previously created block objects.

16. Create the DaRUS dataset object and add the different block objects to it:

```
[ ]: dataset.add_metadatablock(citation_block)
     dataset.add_metadatablock(process_block)
     dataset.add_metadatablock(engineering_block)
```

In order to create an archive following the OMEX standard, the `archive` and `VCard` objects need to be created first. As currently undefinied Internet Media Types, AnIML and PDH are created as application/x types and added to the known formats of *python-libcombine* before continuing to add the AnIML document to the archive object.

1. Create the archive object and VCard objects:

```
[ ]: archive = CombineArchive()
```

```
[ ]: list_of_VCards = []
for creator in citation_block.author:
    new_VCard = VCard()
    new_VCard.family_name = creator.name.split(", ")[0]
    new_VCard.given_name = creator.name.split(", ")[1]
    new_VCard.organization = creator.affiliation
    list_of_VCards.append(new_VCard)
```

3. Add the AnIML document to the archive object:

```
[ ]: local_path = str(path_to_AnIML_file)
archive_path = f"./{str(path_to_AnIML_file.name)}"
format_check = KnownFormats.lookupFormat("animl")
is_master = True
```

```
[ ]: _void = archive.addFile(local_path, archive_path, format_check, is_master)
del _void
```

In the following steps, a description object containing several descriptions, dates, and author information is created. This is then added to the archive object. Optionally, additional files (e.g., PDH, TSV, PNG) can be added to the archive object, as well. Finally, the archive object is serialized to OMEX or ZIP format.

11. Serialize the archive object to OMEX or ZIP format:

```
[ ]: _void = archive.writeToFile(str(path_to_datasets / f"processed/fairsaxs_220512.zip"))
del _void
```

The created OMEX archive can then be uploaded to DaRUS. The Notebook additionally enables downloading and editing of DaRUS datasets.

Upload to DaRUS

The upload of a draft to a DaRUS repository requires an API token with appropriate permissions. The URL of the Dataverse, as well as the API token are inferred from the environment variables.

1. Add unpacked archive to dataset object for upload to DaRUS:

```
[ ]: dataset.add_file(dv_path="fairsaxs_220502.zip", local_path=str(path_to_datasets / "processed/fairsaxs_220502.zip"))
```

2. Upload to DaRUS by stating the target repository and the file or directory to be uploaded and print the resulting DOI:

```
[ ]: p_id = dataset.upload("sfb1333-giesselmann-bruckner")
print(p_id)
```
